# P-1278. Activity of cefiderocol and comparator agents against carbapenem-resistant Enterobacterales, including molecularly characterized isolates recovered from United States hospitals in 2020–2024

**DOI:** 10.1093/ofid/ofaf695.1468

**Published:** 2026-01-11

**Authors:** Mariana Castanheira, John Kimbrough, Joshua Maher, Rodrigo E Mendes

**Affiliations:** Element, North Liberty, IA; Element Iowa City (JMI Laboratories), North Liberty, Iowa; Element Materials Technology/Jones Microbiology Institute, North Liberty, Iowa; Element Iowa City (JMI Laboratories), North Liberty, Iowa

## Abstract

**Background:**

Carbapenem-resistant Enterobacterales (CRE) are often multidrug resistant and limited therapeutic options are available to treat infections caused by these isolates. Cefiderocol is a siderophore cephalosporin that is stable to hydrolysis by β-lactamases, including serine-carbapenemases and metallo-β-lactamases that can be present in CRE isolates. We evaluated the activity of cefiderocol and comparator agents against CRE isolates collected in US hospitals from 2020-2024.

**Figure:**
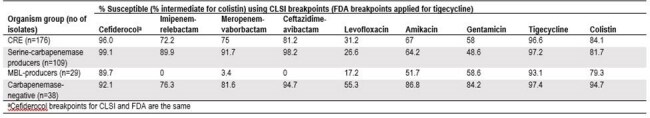

**Methods:**

18,597 Enterobacterales were collected from 35 US hospitals during 2020–2024. Susceptibility testing was performed by the CLSI reference broth microdilution method using cation-adjusted Mueller-Hinton broth and iron-depleted cation-adjusted Mueller-Hinton broth for cefiderocol. CLSI and/or FDA breakpoints were applied. CRE isolates (resistant to imipenem and/or meropenem) were screened for β-lactamase genes using whole genome sequencing.

**Results:**

176 (0.9% overall) isolates were CREs, consisting of 76 *K. pneumoniae* isolates and 100 isolates from 9 other species/complexes. These isolates were observed in all US Census Divisions, except West North Central, with percentages ranging from 0.3% in the Northeast to 2.0% in the Mid-Atlantic. Serine-carbapenemase-encoding genes were detected in 109 isolates, being *bla*_KPC-2_ (n=40) and *bla*_KPC-3_ (n=52) the most common. MBL-encoding genes were observed in 29 isolates and *bla*_NDM-1_ noted in 13 isolates alone or with other carbapenemases. 38 isolates carried no carbapenemase genes. Cefiderocol inhibited 96.0% of the CREs at the CLSI/FDA breakpoint (Figure). Cefiderocol was active against 99.1%, 89.7% and 92.1% of the isolates producing serine carbapenemases, MBLs and carbapenemase-negative isolates, respectively. β-lactam/β-lactamase inhibitor combinations had limited activity against MBL isolates.

**Conclusion:**

Cefiderocol displayed activity against most CRE isolates, including isolates carrying MBL genes that are resistant to multiple comparator agents. Cefiderocol should be considered as an important option for the treatment of infections caused by these resistant organisms.

**Disclosures:**

Mariana Castanheira, PhD, Melinta Therapeutics: Advisor/Consultant|Melinta Therapeutics: Grant/Research Support Rodrigo E. Mendes, PhD, GSK: Grant/Research Support|Shionogi & Co., Ltd.: Grant/Research Support|United States Food and Drug Administration: FDA Contract Number: 75F40123C00140

